# Repeatability and sensitivity to change of non-invasive end points in PAH: the RESPIRE study

**DOI:** 10.1136/thoraxjnl-2020-216078

**Published:** 2021-02-25

**Authors:** Andrew J Swift, Frederick Wilson, Marcella Cogliano, Lindsay Kendall, Faisal Alandejani, Samer Alabed, Paul Hughes, Yousef Shahin, Laura Saunders, Charlotte Oram, David Capener, Alex Rothman, Pankaj Garg, Christopher Johns, Matthew Austin, Alistair Macdonald, Jo Pickworth, Peter Hickey, Robin Condliffe, Anthony Cahn, Allan Lawrie, Jim M Wild, David G Kiely

**Affiliations:** 1 Department of Infection, Immunity and Cardiovascular Disease, The University of Sheffield, Sheffield, UK; 2 Research and Development, GlaxoSmithKline, Stevenage, Hertfordshire, UK; 3 Sheffield Pulmonary Vascular Disease Unit, Royal Hallamshire Hospital, Sheffield, UK; 4 Department of Respiratory Medicine, Bedford Hospital, Bedford, UK

**Keywords:** primary pulmonary hypertension, imaging/CT MRI etc

## Abstract

End points that are repeatable and sensitive to change are important in pulmonary arterial hypertension (PAH) for clinical practice and trials of new therapies. In 42 patients with PAH, test–retest repeatability was assessed using the intraclass correlation coefficient and treatment effect size using Cohen’s d statistic. Intraclass correlation coefficients demonstrated excellent repeatability for MRI, 6 min walk test and log to base 10 N-terminal pro-brain natriuretic peptide (log_10_NT-proBNP). The treatment effect size for MRI-derived right ventricular ejection fraction was large (Cohen’s d 0.81), whereas the effect size for the 6 min walk test (Cohen’s d 0.22) and log_10_NT-proBNP (Cohen’s d 0.20) were fair. This study supports further evaluation of MRI as a non-invasive end point for clinical assessment and PAH therapy trials.

Trial registration number NCT03841344.

## Introduction

Pulmonary arterial hypertension (PAH) is progressive, leading to right ventricular (RV) failure and death.^1^ Accurate measurement of RV function is important for assessment of disease severity and prognosis.^2–4^ Despite new therapies and improvements in survival,^5^ PAH remains a life-shortening condition. MRI is the gold standard for RV assessment,^6^ has prognostic value^2^ and predicts clinical worsening^7^ in PAH. A trial end point that is highly repeatable, is sensitive to treatment and predicts outcomes would be highly desirable.^8 9^ MRI has been proposed as a trial end point in PAH,^8 9^ however, there is limited data on repeatability and treatment effect size.

## Methods

### Patients

Patients with PAH who were treatment-naïve commencing therapy, prevalent undergoing escalation of therapy and clinically stable requiring no escalation of therapy, were recruited. See online supplemental file S1.

10.1136/thoraxjnl-2020-216078.supp1 Supplementary data

### Study investigations

Investigations performed at visit 1 included N-terminal pro-brain natriuretic peptide (NT-ProBNP), 6 min walk test (6MWT) and MRI. Follow-up visits 2 and 3 occurred approximately 6 months after study visit 1. Visits 2 and 3 occurred within 24 hours of each other (online supplemental figure S2).

10.1136/thoraxjnl-2020-216078.supp2 Supplementary data

### MRI acquisition and analysis

All MRI examinations were performed on either a 1.5 T GE HDx (GE Healthcare, Milwaukee, USA) whole body scanner using an 8-channel cardiac coil or a 3 T Philips Ingenia (Best, The Netherlands) whole body scanner using a 32-channel dStream torso coil (online supplemental file S1). Analysis of MRI was undertaken blinded to the patient’s data. RV parameters and pulmonary arterial flow were analysed on Qmass MEDIS suite (V.3.0.18.0, Medical Imaging Systems, The Netherlands) on short axis and phase contrast images, respectively. Regions of interest were drawn on the pulmonary artery and left atrium of the dynamic contrast-enhanced perfusion images to calculate first pass pulmonary transit time and full width at half maximum using in-house software (see online supplemental figure S3).

10.1136/thoraxjnl-2020-216078.supp3 Supplementary data

### Six min walk test and NT-ProBNP

The 6MWT was performed by a respiratory physiologist. NT-ProBNP analysis was performed on patient plasma samples using the Luminex 100/200 multiplex analyser using the cardiovascular marker kit (HCVD1MAG-67K Millipore) at the end of the study.

### Statistical analysis

Repeatability was determined by the intraclass correlation coefficient (ICC) using a two-way mixed absolute agreement model with the average measure recorded. An ICC of ≥0.75 was considered excellent, 0.60–0.74 good, 0.40–0.59 fair and <0.40 poor. Mean difference and 95% CIs were presented where appropriate. Cohen’s d (calculated with the averaged SD, d_av_) was used to assess the standardised treatment effect size between visit 1 and visit 2.^10^ A Cohen’s d value of <0.20 was considered no change, 0.20–0.49 was considered fair change, 0.50–0.79 was considered a medium change and ≥0.80 was considered a large change. All analysis was performed on SPSS V.22 and GraphPad Prism V.16.

## Results

### Patients

Of 42 patients who completed the study, 16 were incident and treatment-naïve and initiated PAH therapy, 12 were prevalent and underwent an escalation of therapy and 14 were stable on therapy with no change in treatment occurring between the study visits.(online supplemental table S5).

### Test–test repeatability (visits 2 and 3)

In patients with PAH, test–test repeatability was assessed between visits 2 and 3; 6MWT (ICC 0.987) and log_10_NT-ProBNP (ICC 0.772) had excellent repeatability. Of cardiac MRI metrics (table 1), all showed excellent repeatability. Data for MRI pulmonary flow and perfusion transit times are shown in table 1.

**Table 1:** Repeatability in all patients with PAH (ICC), and treatment effect size for patients with PAH initiating or escalating PAH therapy

	All PAH		Patients with PAH initiating or escalating therapy										
	N	ICC	N	Visit 1		Visit 2		Change (Visit 1–visit 2)			95% CI		Cohen’s d
				Mean	SD	Mean	SD	Mean difference	SD	SEM	Lower	Upper	
**Walk test**													
6MWT distance (m)	39	0.987	24	325.63	156.30	361.50	166.29	−35.88	79.06	16.14	−69.26	−2.49	0.22
**Blood tests**													
Log NT-ProBNP	32	0.772	24	2.76	0.46	2.67	0.41	0.09	0.32	0.07	−0.05	0.22	0.20
**MRI metrics**													
***SA with threshold***													
RVEDM (g)	40	0.970	26	117.80	45.72	99.40	43.96	18.40	30.90	6.06	5.92	30.88	0.41
RVESM (g)	40	0.980	26	106.68	39.73	94.61	42.08	12.06	26.79	5.25	1.24	22.88	0.29
RVEDV (mL)	40	0.969	26	145.71	39.12	146.03	55.87	−0.32	29.13	5.71	−12.08	11.45	0.01
RVESV (mL)	40	0.983	26	93.93	34.66	81.28	41.40	12.65	22.02	4.32	3.76	21.55	0.33
RVEF (%)	40	0.883	26	36.56	11.48	45.69	11.12	−9.12	10.45	2.05	−13.35	−4.90	0.81
RVSV (mL)	40	0.864	26	51.78	17.30	64.75	23.92	−12.97	23.27	4.56	−22.37	−3.57	0.62
RVCO (L/min)	40	0.886	26	3.95	1.45	4.48	1.55	−0.53	1.54	0.30	−1.15	0.09	0.35
Systolic septal angle (^o^)	40	0.852	27	163.33	16.45	156.81	14.00	6.52	11.28	2.17	2.06	10.98	0.43
Diastolic septal angle (^o^)	40	0.897	27	153.11	14.73	145.48	10.44	7.63	10.15	1.95	3.61	11.65	0.60
**Q flow**													
Net flow volume (mL)	41	0.893	26	58.05	30.18	69.49	31.30	−11.44	34.83	6.83	−25.51	2.62	0.37
Forward flow volume (mL)	41	0.860	26	60.37	27.58	72.33	29.15	−11.96	31.97	6.27	−24.88	0.95	0.42
Backward flow volume (mL)	41	0.817	26	2.32	6.76	2.84	5.74	−0.52	5.85	1.15	−2.88	1.85	0.08
Regurgitant fraction (%)	41	0.731	26	6.28	19.58	5.42	11.52	0.87	18.77	3.68	−6.71	8.45	0.05
Average flow velocity (cm/s)	41	0.909	26	7.31	3.60	8.25	3.69	−0.94	3.69	0.72	−2.43	0.55	0.26
Peak flow velocity (cm/s)	41	0.582	26	52.97	16.37	67.68	22.71	−14.71	19.35	3.79	−22.53	−6.90	0.74
Diastolic vessel area (mm^2^)	41	0.933	26	981.10	257.92	961.84	242.96	19.26	104.52	20.5	−22.96	61.48	0.08
Systolic vessel area (mm^2^)	41	0.953	26	1077.57	279.96	1083.62	266.78	−6.05	101.08	19.82	−46.88	34.77	0.02
Pulmonary arterial pulsatility (%)	41	0.776	26	9.96	4.87	13.00	5.12	−3.04	3.62	0.71	−4.50	−1.58	0.61
***DCE imaging***													
Pulmonary transit time (s)	36	0.728	21	6.76	1.81	6.12	1.88	0.64	1.60	0.35	−0.09	1.37	0.35
FWHM (s)	32	0.906	18	7.89	3.14	6.20	2.40	1.68	2.19	0.52	0.60	2.77	0.60

Data are shown for all patients with PAH initiating or escalating PAH therapy.

DCE, dynamic contrast-enhanced imaging; FWHM, full width at half maximum; ICC, intraclass correlation coefficient; Log_10_NT-ProBNP, log to base 10 N-terminal pro-brain natriuretic peptide; 6MWT, six min walk test; PAH, pulmonary arterial hypertension; RVCO, right ventricular cardiac output; RVEDM, right ventricle end-diastolic mass; RVEDV, right ventricle end-systolic volume; RVEF, right ventricle end-systolic volume; RVESM, right ventricle end-systolic mass; RVSV, right ventricle stroke volume; SA, short axis.

### Treatment effect size (visits 1 and 2)

**Figure 1:**
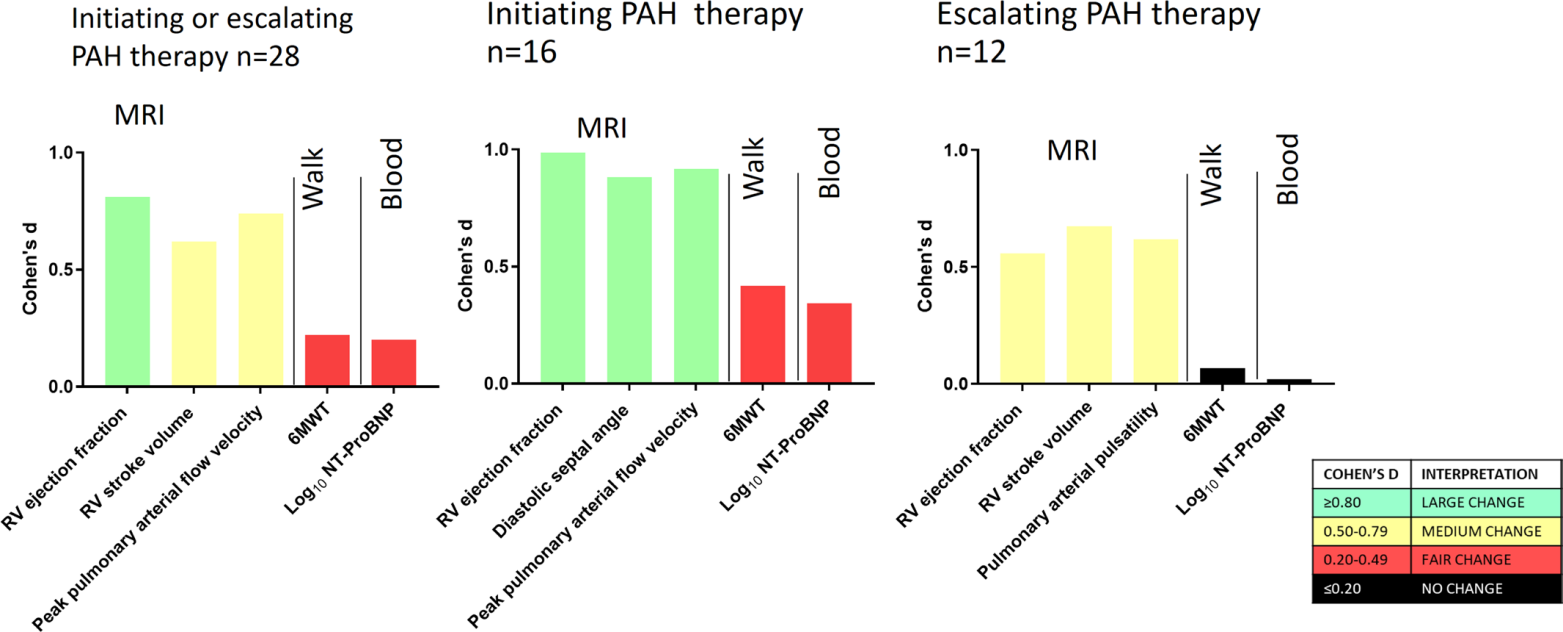
Comparison of treatment effect size using Cohen’s d results in patients initiating and/or escalating pulmonary arterial hypertension (PAH) therapy. 6MWT, 6 min walk test; Log_10_NT-ProBNP, log to base 10 N-terminal pro-brain natriuretic peptide; RV, right ventricular.

**Figure 2:**
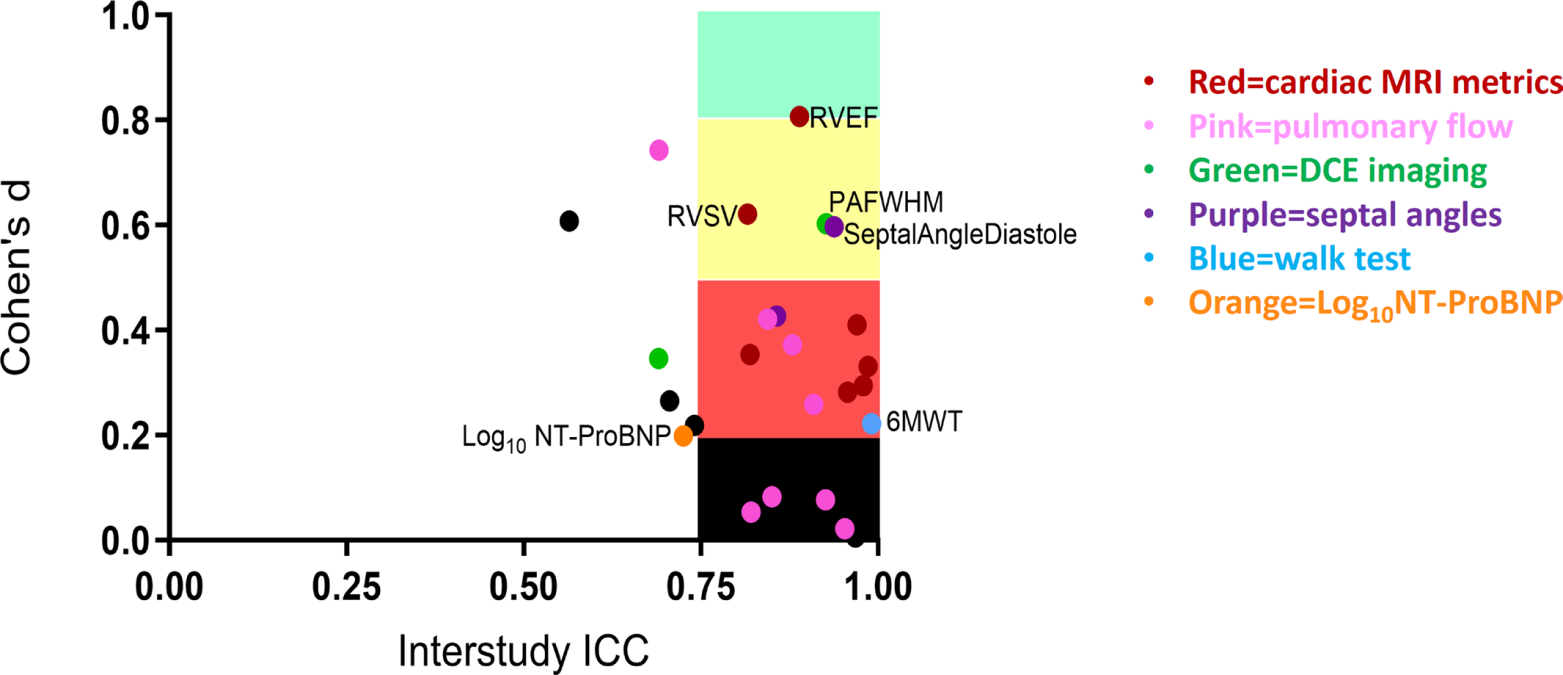
Cohen’s d versus interstudy intraclass correlation coefficient (ICC) for study measurements. DCE, dynamic contrast-enhanced imaging; Log_10_NT-ProBNP, log to base 10 N-terminal pro-brain natriuretic peptide; PAFWHM, pulmonary arterial full width at half maximum; RVEF, right ventricular ejection fraction; RVSV, right ventricle stroke volume; 6MWT 6 min walk test. ICC >0.75=excellent repeatability. Cohen’s d value of <0.20 was considered no change, 0.20–0.49 was considered fair change, 0.50–0.79 was considered a medium change and ≥0.80 was considered a large change.

For all patients, initiating or escalating therapy (n=28), the only measurement with a large treatment effect size was RV ejection fraction (Cohen’s d 0.81). The 6MWT (Cohen’s d 0.22) and NT-ProBNP (Cohen’s d 0.20) demonstrated a fair treatment effect size (table 1). Figure 1 shows Cohen’s d values for the top three MRI end points, the 6MWT and NT-proBNP. Figure 2 shows ICC versus Cohen’s d value for all end points. In patients initiating PAH therapy, RV ejection fraction (Cohen’s d 0.99), diastolic septal angle (Cohen’s d 0.88) and peak pulmonary arterial flow velocity (Cohen’s d 0.92) had a large treatment effect size. In patients escalating therapy, RV ejection fraction, RV stroke volume and pulmonary arterial pulsatility had a medium effect size, whereas NT-ProBNP (Cohen’s d 0.02) and 6MWT (Cohen’s d 0.07) demonstrated no treatment effect (see online supplemental figure S4). The stable patient group showed either no or fair changes across all measured parameters (online supplemental table S6).

10.1136/thoraxjnl-2020-216078.supp4 Supplementary data

## Discussion

Investigations used to monitor disease severity in patients with PAH, namely 6MWT distance, NT-ProBNP level and MRI metrics, had excellent repeatability. In contrast, only MRI (RVEF) demonstrated a large treatment effect size in patients initiating or escalating therapies, whereas for the 6MWT and NT-ProBNP the treatment effect sizes were fair.

As observed in previous clinical trials^1^ and highlighted at the 6th World Symposium,^9^ all metrics evaluated in patients with PAH escalating therapy had a lower treatment effect size compared with treatment-naïve patients initiating therapy. This represents a challenge when studying the effects of new therapies in PAH where the standard of care is combination treatment.^1^ Importantly, MRI was still able to detect a medium treatment effect size in patients receiving background PAH therapy. Due to the large cost of conducting PAH therapy trials, strategies to reduce the size of studies and their duration using a surrogate end point that is repeatable and has a large treatment effect size would be highly desirable.^9^

This study has a number of limitations including the small sample size and the lack of comparison with invasively measured pulmonary haemodynamics. Nonetheless, we have demonstrated in this exploratory study that MRI, the gold standard for RV function assessment, detects a larger treatment effect than the 6MWT or NT-proBNP. This may reflect the ceiling effect of the 6MWT and the effect of comorbidities (including chronic kidney disease) that may influence 6MWT distance and NT-proBNP levels. MRI metrics predict clinical worsening^7^ and mortality^2–4^ fulfilling many of the criteria of a surrogate end point.^9^ Given that pulmonary haemodynamics are commonly used in early phase PAH studies,^1^ a direct comparison of MRI metrics and pulmonary haemodynamics, to detect longitudinal change following PAH therapy, is now required if MRI imaging is to be considered a primary end-point for PAH therapy trials.^8 9^

This study demonstrates the high repeatability of MRI metrics in PAH and the large treatment effect size support further evaluation of MRI as a non-invasive endpoint in PAH therapy trials.
