# Supplementary material for: Repeatability and sensitivity to change of non-invasive end points in PAH: the RESPIRE study
Source: Thorax. 2021 Feb 25;76(10):1032–5. doi: 10.1136/thoraxjnl-2020-216078 (PMC8461450; doi:10.1136/thoraxjnl-2020-216078)
Supplement: Supplementary data [file thoraxjnl-2020-216078supp001.pdf]

Supplementary file S1

Methods

Patients

Patients with PAH were prospectively recruited from a nationally designated pulmonary hypertension referral centre. We aimed to recruit 3 approximately equal size groups of patients: incident treatment-naïve patients commencing therapy, prevalent patients escalating therapy and patients who were deemed to be clinically stable and required no escalation of therapy. For study inclusion, patients were required to have idiopathic pulmonary arterial hypertension (IPAH), heritable PAH, PAH associated with connective tissue disease or portal hypertension, to have undergone RHC and have a mean pulmonary arterial pressure ≥ 25 mmHg and pulmonary arterial wedge pressure ≤ 15 mmHg and to have had other causes of pulmonary hypertension excluded. Exclusion criteria included pregnancy, allergy to contrast medium, contraindication to MRI or known active Hepatitis B or C or HIV infection. Ethical and institutional review board approval was obtained and all patients provided informed written consent.

Study investigations

Investigations performed at visit 1 included a blood draw (NT-ProBNP), an exercise test (6MWT) and MRI. Any subsequent treatment initiation or escalation occurred after visit 1 investigations were completed. Follow-up visits 2 and 3 occurred approximately 6 months after study visit 1. Visits 2 and 3 occurred within 24 hours of each other. Typically, visit 2 investigations occurred in the morning, and visit 3 investigations occurred in the afternoon or the following day. The study flow diagram, **Supplementary figure S2**, describes the study investigations. Patients took their usual medications approximately 1 hour prior to their MRI. Patients rested for 45 minutes after the 6-minute walking test to allow for recovery as per ATS recommendations. Patients were asked to refrain from exercise, caffeinated drinks and alcohol between investigations during their visits. Follow up scans at visits 2 and 3 were performed on the same MRI system as at visit 1.

MRI acquisition

The rationale for performing measurements on two different MRI scanners at different field strengths and from different vendors was to assess the variation introduced in the candidate MRI measurements across platforms. Short axis cine cardiac imaging stacks were performed using a balanced single shot free precession sequence with full ventricular coverage. End-systole was defined as the smallest chamber area on each slice and end-diastole was defined as the first cine phase of the R-wave triggered acquisition. The phase-contrast velocity imaging was performed 1cm above and orthogonal to the pulmonary valve. For dynamic contrast enhanced perfusion imaging a three-dimensional spoiled gradient echo time-resolved Magnetic Resonance Angiography sequence was used, positioned coronal across the chest with full lung coverage with an intravenous bolus injection of Gadovist (Bayer Healthcare, Germany) (0.05 mL/kg at 4ml/s followed by a saline flush of 20mL). Sequence parameters were: TR=2.1ms, T=0.7ms, FA=30°, BW=250kHz, with a reconstructed voxel size of 1.875x1.875x10mm and an effective frame rate of ~0.5s per whole lung volume.

MRI analysis

Epicardial and endocardial contours were drawn using semi-automatic tools with intensity thresholding of the structures within the endocardial contour, and biventricular volume, mass and function recorded. Regions of interest were drawn on the pulmonary artery and left atrium of the dynamic contrast enhanced perfusion images to calculate first pass pulmonary transit time (PTT) and full-width-half-maximum (FWHM) using in-house software. See **Supplementary figure S3**

Statistical analysis

Following histogram analysis, variables were categorised into normal and non-normal distributions: if normally distributed, mean and standard deviation were presented, whereas if non-normally distributed, median and interquartile range were presented.

Results

Patients

A total of 104 patients with PAH or suspected PAH were approached to take part in the study, 58 patients with suspected PAH were consented; of the 58 patients, 44 met the entry criteria after completion of their baseline study investigations and 42 completed the study. Patients had a mean age of 51 years and 83% were female. **Supplementary Table S5** presents demographic, pulmonary function test and right heart catheter data for all patients. Two patients experienced some claustrophobic symptoms at both visits but completed the study protocol. The mean time intervals between baseline and follow up for these 3 subgroups were: 6 months (standard deviation (SD) 3 months), 6 months (SD 1 month), and 8 months (SD 2 months) respectively. MRI and 6MWT were normally distributed on visual inspection of histograms. NT-ProBNP had a skewed distribution, however, following $\log_{10}$ transformation the distribution was normalised.

Supplementary figures

S2 Study flow chart illustrating the three study visits and mandatory investigations. All patients and healthy volunteers included in the study underwent the same protocol

S3 Illustration of the diastolic (A) and systolic (B) short axis cardiac cine MRI (epicardial and endocardial contours drawn and thresholding applied). Magnitude (C) and flow (D) images from phase contrast MRI sequence placed orthogonal to the main pulmonary artery. Dynamic contrast enhanced images illustrating contrast in the pulmonary arteries (E) and passing through to the left atrium, left ventricle and aorta (F).

S4 Line plots showing the changes in right ventricular ejection fraction (RVEF) and right ventricular stroke volume (RVSV) for individual patients in the combined initiating and escalating therapy group.

Supplementary tables

Supplementary table S5: Demographics, therapy, diagnostic and right heart catheter data in patients with PAH.

	All patients n=42	Treatment naïve n=16	Treatment change n=12	Stable n=14
**Demographics**				
Age (yrs)	51 (15)	54 (14)	52 (16)	46 (15)
Female [n (%)]	35 (83)	14 (88)	10 (83)	11 (79)
WHO functional class	I (0) II (2) III (35) IV (5)	I (0) II (0) III (13) IV (3)	I (0) II (0) III (11) IV (1)	I (0) II (2) III (11) IV (1)
**Treatment (visit 1 therapy)**				
Monotherapy	10 (24%)	0 (0%)	8 (67%)	2 (14%)
Combination therapy	10 (24%)	0 (0%)	4 (33%)	6 (43%)
Parenteral prostanoid	6 (14%)	0 (0%)	0 (0%)	6 (43%)
Calcium channel blocker	0 (0%)	0 (0%)	0 (0%)	0 (0%)
**Treatment (visit 2 therapy)**				
Monotherapy	4 (10%)	1 (6%)	1 (8%)	2 (14%)
Combination oral therapy	23 (54%)	9 (56%)	8 (67%)	6 (43%)
Parenteral prostanoid	13 (31%)	4 (25%)	3 (25%)	6 (43%)
Calcium channel blocker	2 (5%)	2 (13%)	0 (0%)	0 (0%)
**Subgroups**				
IPAH and HPAH	25 (60.9%)	8 (50%)	8 (66.6%)	10 (71.1%)
PAH CTD	10 (24.4%)	6 (37.5%)	2 (16.6%)	2 (14.3%)
PAH congenital	1 (2.4%)	1 (6.25%)	0 (0%)	0 (0%)
PAH portal	4 (10%)	1 (6.25%)	1 (8.3%)	2 (14.3%)
PAH drug and toxins	1 (2.4%)	0 (0%)	1 (8.3%)	0 (0%)
**Walk test**				
6MWT Distance (m)	375 (167)	303 (144)	332 (179)	488 (120)
**Blood tests**				
Log_{10}NT-ProBNP*	561 (258 to 906)	619 (213 to 1095)	665 (301 to 1356)	386 (176 to 606)
**Right heart catheter**				
mRAP (mmHg)	11 (7)	13 (7)	14 (7)	6 (2)
mPAP (mmHg)	52 (10)	53 (7)	58 (10)	46 (14)
PCWP (mmHg)	10 (3)	10 (2)	12 (2)	10 (3)
CI (L/min/m2)	2.4 (0.9)	2.0 (0.8)	2.0 (0.7)	3.0 (0.8)
PVR (Wood units)	11 (6)	14 (6)	13 (5)	7 (5)
Mixed venous oxygen saturations (%)	64 (10)	60 (11)	64 (6)	69 (7)

Values are mean (SD) or n (%) unless otherwise stated
* Median (IQR) presented

Supplementary table S6 Mean and standard deviation of walk test, NT-ProBNP and MRI metrics in stable patients with PAH who do have no change in PAH therapy between visit 1 or visit 2.

	N	Visit 1		Visit 2		Change (Visit 1 – Visit 2)			95% Confidence Interval		Cohen's d
		Mean	SD	Mean	SD	Mean difference	SD	SEM	Lower	Upper	
**Walk test**											
6MWT Distance (m)	12	479.83	121.47	473.50	117.98	6.33	80.62	23.27	-44.89	57.56	0.05
**Blood test**											
Log NT-ProBNP	14	2.53	0.44	2.67	0.32	-0.14	0.37	0.10	-0.35	0.08	0.36
**SA with threshold**											
RVEDM (g)	14	85.34	27.13	82.55	26.41	2.79	10.47	2.80	-3.25	8.83	0.10
RVESM (g)	14	72.00	24.77	75.33	30.28	-3.33	13.21	3.53	-10.96	4.30	0.12
RVEDV (ml)	14	97.89	31.92	105.75	41.23	-7.86	23.09	6.17	-21.19	5.47	0.21
RVESV (ml)	14	49.18	20.47	52.07	19.88	-2.89	9.26	2.47	-8.24	2.45	0.14
RVEF (%)	14	50.45	8.51	49.58	11.01	0.87	13.54	3.62	-6.95	8.69	0.09
RVSV (ml)	14	48.70	15.29	53.67	26.94	-4.97	25.42	6.79	-19.65	9.71	0.23
RVCO (L/min)	14	3.27	1.12	3.60	2.06	-0.33	1.74	0.47	-1.34	0.68	0.20
Systolic septal angle (°)	13	149.69	16.24	153.00	12.01	-3.31	14.44	4.00	-12.03	5.42	0.23
Diastolic septal angle(°)	13	142.31	10.56	143.15	9.11	-0.85	5.76	1.60	-4.32	2.63	0.09
**Q flow**											
Net flow volume (ml)	13	79.94	55.65	72.38	32.66	7.56	45.72	12.68	-20.07	35.18	0.17
Forward flow volume (ml)	13	82.17	53.54	74.39	30.50	7.78	43.20	11.98	-18.33	33.89	0.18
Backward flow volume (ml)	13	2.24	5.05	2.02	3.83	0.22	4.85	1.35	-2.71	3.15	0.05
Regurgitant fraction (%)	13	5.67	12.77	4.87	10.24	0.81	12.74	3.53	-6.89	8.51	0.07
Average flow velocity (cm/s)	13	7.97	4.10	8.74	4.57	-0.78	3.00	0.83	-2.59	1.04	0.18
Peak flow velocity (cm/2)	13	66.06	32.37	69.33	29.30	-3.27	39.47	10.95	-27.12	20.58	0.11
Diastolic vessel area (mm2)	13	988.46	314.83	916.21	290.08	72.26	241.04	66.85	-73.40	217.91	0.24
Systolic vessel area (mm2)	13	1110.66	305.79	1042.87	287.10	67.78	245.12	67.98	-80.34	215.91	0.23
Pulmonary arterial pulsatility (%)	13	13.98	6.78	15.25	7.02	-1.27	5.42	1.50	-4.55	2.01	0.18
**DCE imaging**											
Pulmonary Transit Time (s)	11	5.67	1.13	5.91	1.67	-0.25	1.34	0.40	-1.15	0.65	0.17
FWHM (s)	11	5.21	1.68	5.02	1.50	0.20	1.66	0.50	-0.92	1.31	0.12

6MWT= six minute walk test, Log10NT-ProBNP=log to base 10 N Terminal brain naturitic peptide, SA= short axis, RVEDM=right ventricle end-diastolic mass, RVESM=right ventricle end-systolic mass, RVEDV=right ventricle end-systolic volume, RVEF=right ventricle end-systolic volume, RVSV=right ventricle stroke volume, RVCO= right ventricular cardiac output, DCE= dynamic contrast enhanced imaging, FWHM=full-width half maximum

Swift AJ, *et al. Thorax* 2021; 76:1032–1035. doi: 10.1136/thoraxjnl-2020-216078
