# Supplementary figures and images for: Repeatability and sensitivity to change of non-invasive end points in PAH: the RESPIRE study
Source: Thorax. 2021 Feb 25;76(10):1032–5. doi: 10.1136/thoraxjnl-2020-216078 (PMC8461450; doi:10.1136/thoraxjnl-2020-216078)

# RESPIRE study: Sensitivity and repeatability of non-invasive endpoints

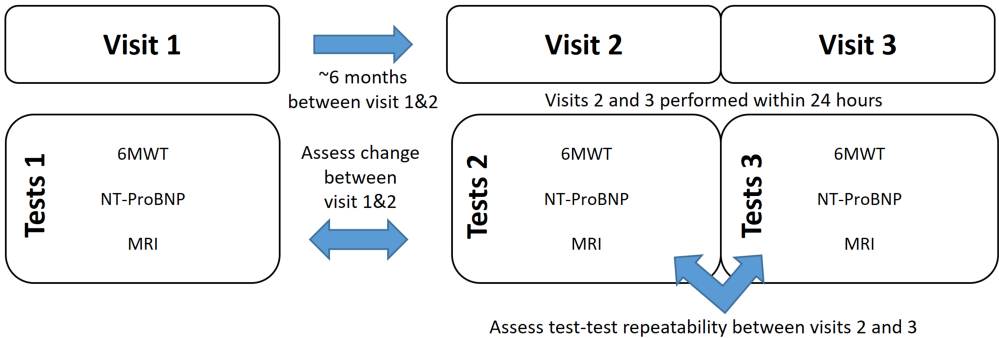

Supplement: Supplementary data [file thoraxjnl-2020-216078supp002.pdf]

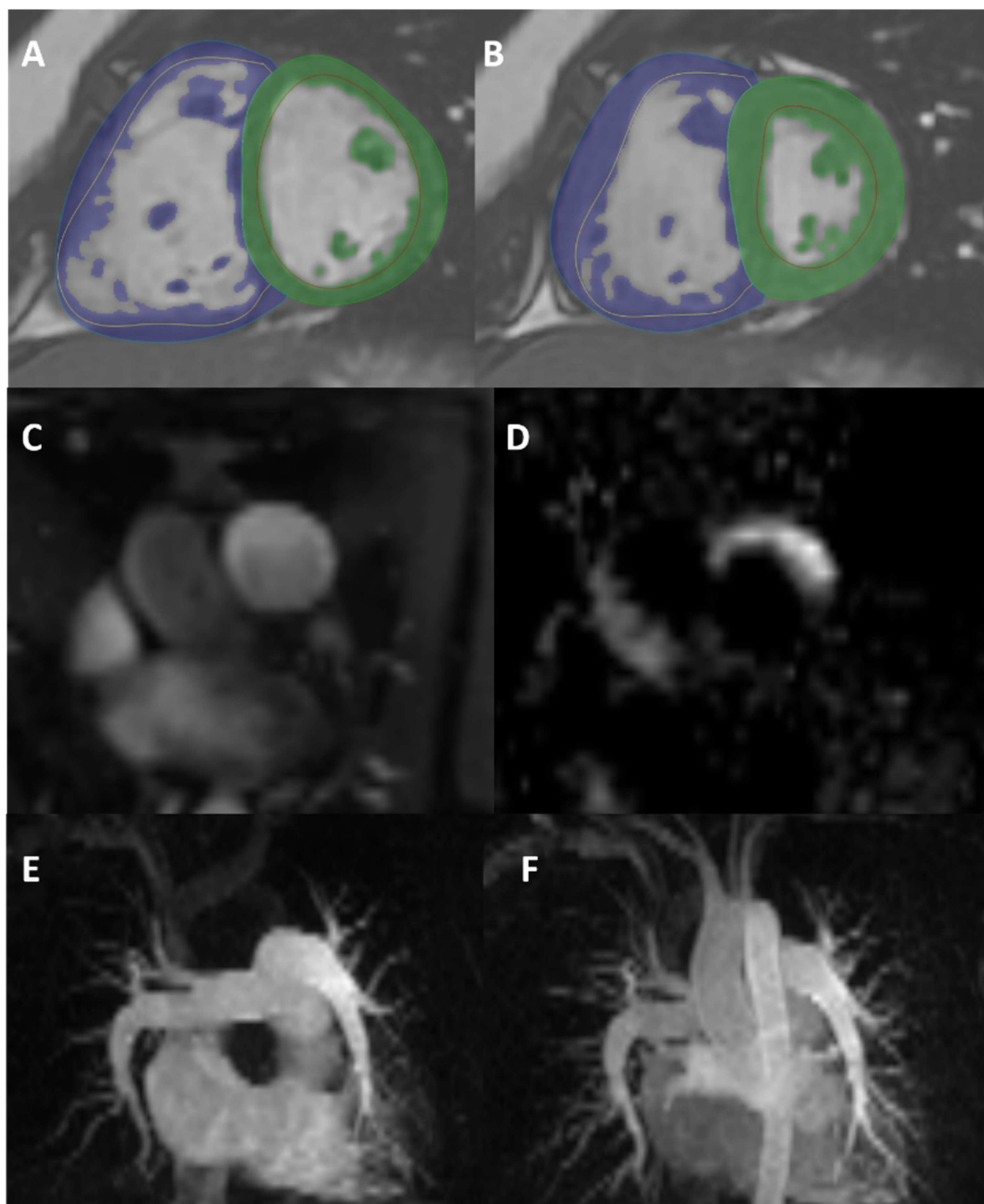

Supplement: Supplementary data [file thoraxjnl-2020-216078supp003.pdf]

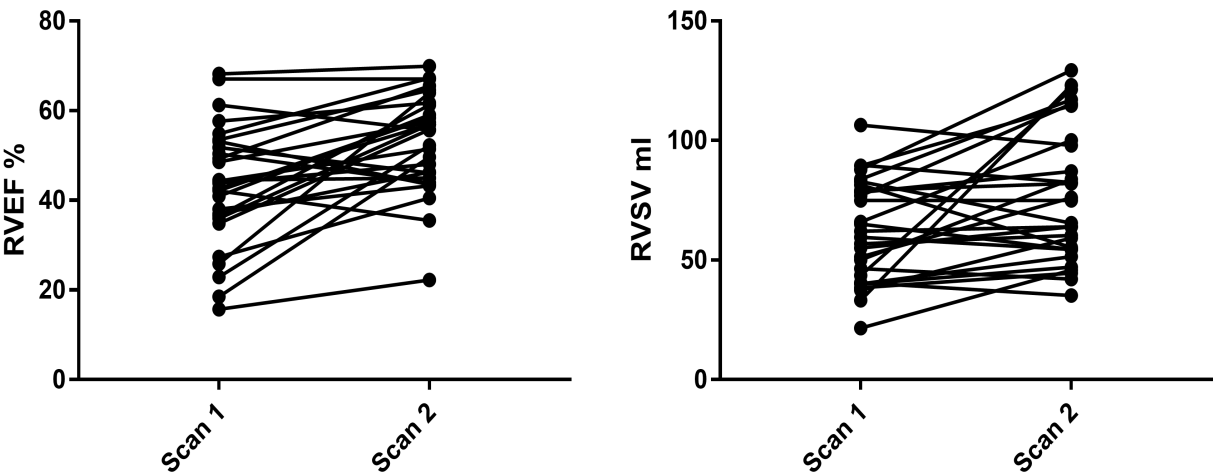

Supplement: Supplementary data [file thoraxjnl-2020-216078supp004.pdf]
